# Editorial: Patient safety revisited

**DOI:** 10.1120/jacmp.v12i2.3625

**Published:** 2011-05-16

Once again a recent article in ${\text{The New York Times}}^{\left(1\right)}$ has caused angst in the medical physics community because of what appeared to have been incorrect delivery of radiation. Following reporting of the incident, many email messages appeared on list servers written by medical physicists responding to this incident. One of the most vocal of these individuals has been Howard I. Amols, PhD, Chief, Clinical Physics Service, Memorial Sloan Kettering Cancer Center, in New York. Many of the points he raised are worthy of discussion in the clinical medical physics community and for this reason, I invited Dr Amols to express them in a forum that, I hope, will have widespread international readership. The following Guest Editorial, “Can We Really Reduce Error Rates by Medical Physicists?” is presented for your consideration.

Please note that the right side of the viewing window on the JACMP website provides readers with the opportunity to add comments to this Editorial. As well, I would be open to entertaining Guest Editorials with contrary points of view.

George Starkschall, PhD

Editor‐in‐Chief

May 15, 2011

